# Aqua­(2,2′-bipyrimidine-κ^2^ *N*,*N*′)(succin­ato-κ^2^ *O* ^1^,*O* ^4^)copper(II) dihydrate

**DOI:** 10.1107/S1600536809013518

**Published:** 2009-04-18

**Authors:** Xi-Jun Ke, Dong-Sheng Li, Jun Zhao, Qiu-Fen He, Cai Li

**Affiliations:** a College of Mechanical and Material Engineering, Functional Materials Research Institute, China Three Gorges University, Yichang 443002, People’s Republic of China

## Abstract

In the crystal structure of the title compound, [Cu(C_4_H_4_O_4_)(C_8_H_6_N_4_)(H_2_O)]·2H_2_O, the Cu^II^ atom is chelated by a 2,2′-bipyrimidine (bpm) ligand and a succinate anion in the basal plane; a water mol­ecule in the apical position completes the slightly distorted square-pyramidal coordination geometry. Another carboxyl­ate O atom from an adjacent complex is located in the opposite apical direction, with a Cu⋯O distance of 2.706 (3) Å, and is not considered as a bridging atom. Extensive O—H⋯O and O—H⋯N hydrogen bonding is present in the crystal structure.

## Related literature

For general background, see: McCann *et al.* (1997 ▶); Ray *et al.* (2004 ▶); Zhang *et al.* (2004 ▶).
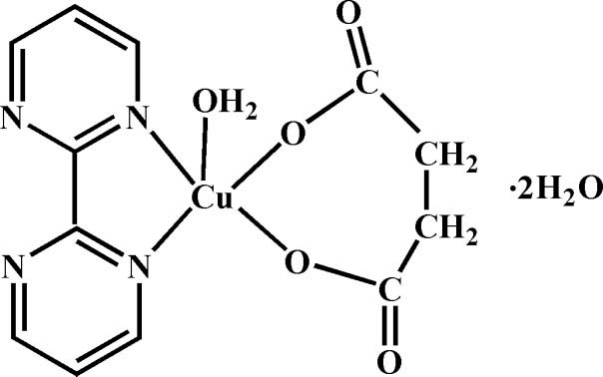


## Experimental

### 

#### Crystal data

[Cu(C_4_H_4_O_4_)(C_8_H_6_N_4_)(H_2_O)]·2H_2_O
*M* *_r_* = 391.83
Monoclinic, 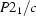

*a* = 10.6905 (8) Å
*b* = 18.9321 (14) Å
*c* = 7.6105 (6) Å
β = 92.2290 (10)°
*V* = 1539.2 (2) Å^3^
*Z* = 4
Mo *K*α radiation
μ = 1.46 mm^−1^
*T* = 293 K
0.30 × 0.20 × 0.09 mm

#### Data collection

Bruker SMART CCD diffractometer
Absorption correction: multi-scan (*SADABS*; Sheldrick, 1996 ▶) *T*_min_ = 0.700, *T*_max_ = 0.877
7725 measured reflections
2735 independent reflections
2085 reflections with *I* > 2σ(*I*)
*R*_int_ = 0.036

#### Refinement

*R*[*F* ^2^ > 2σ(*F* ^2^)] = 0.043
*wR*(*F* ^2^) = 0.123
*S* = 1.06
2735 reflections
235 parameters
10 restraints
H atoms treated by a mixture of independent and constrained refinement
Δρ_max_ = 0.46 e Å^−3^
Δρ_min_ = −0.66 e Å^−3^

### 

Data collection: *SMART* (Bruker, 1997 ▶); cell refinement: *SAINT* (Bruker, 1997 ▶); data reduction: *SAINT*; program(s) used to solve structure: *SHELXTL* (Sheldrick, 2008 ▶); program(s) used to refine structure: *SHELXTL*; molecular graphics: *SHELXTL*; software used to prepare material for publication: *SHELXTL*.

## Supplementary Material

Crystal structure: contains datablocks I, global. DOI: 10.1107/S1600536809013518/xu2509sup1.cif

Structure factors: contains datablocks I. DOI: 10.1107/S1600536809013518/xu2509Isup2.hkl

Additional supplementary materials:  crystallographic information; 3D view; checkCIF report

## Figures and Tables

**Table 1:** Selected bond lengths (Å)

Cu1—O1	2.386 (3)
Cu1—O2	1.918 (3)
Cu1—O5	1.940 (3)
Cu1—N1	2.017 (3)
Cu1—N2	2.012 (3)

**Table 2:** Hydrogen-bond geometry (Å, °)

*D*—H⋯*A*	*D*—H	H⋯*A*	*D*⋯*A*	*D*—H⋯*A*
O1—H1*A*⋯O1*W*	0.85 (4)	1.89 (4)	2.703 (5)	162 (4)
O1—H1*B*⋯O4^i^	0.85 (4)	2.05 (4)	2.903 (4)	178 (4)
O1*W*—H1*WA*⋯O2*W*^ii^	0.85 (4)	1.950 (17)	2.790 (6)	169 (5)
O1*W*—H1*WB*⋯N3^iii^	0.84 (4)	2.45 (5)	3.216 (5)	152 (4)
O1*W*—H1*WB*⋯N4^iii^	0.84 (4)	2.46 (4)	3.130 (5)	137 (5)
O2*W*—H2*WA*⋯O3^iii^	0.85 (4)	2.04 (4)	2.876 (6)	167 (5)
O2*W*—H2*WB*⋯O3^iv^	0.85 (4)	1.94 (4)	2.777 (5)	168 (4)

Symmetry codes: (i) 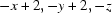; (ii) 
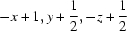
; (iii) 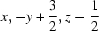; (iv) 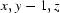.

**Fig. 1.**
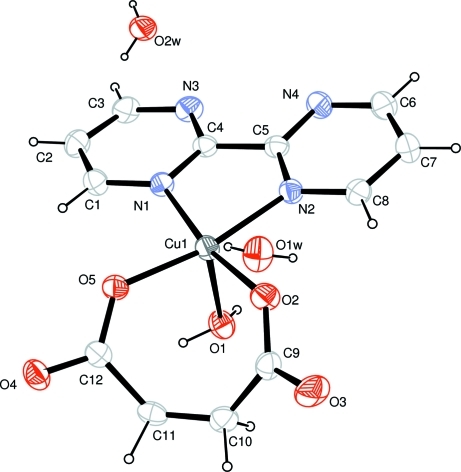
View of (I) showing the atom-labelling scheme. Displacement ellipsoids are drawn at the 30% probability level.

## supplementary crystallographic information

### Comment

Recently, the area of metal-organic framework materials has become one of the intense research activities for their fascinating structural diversities and potential applications in catalysis, nonlinear optics and molecular sensing. As an important family of multidentate O-donor ligands, saturated aliphatic carboxylate ligands have been extensively employed in the preparation of metal-organic complexes because of their potential properties and intriguing structural topologies (McCann *et al.*, 1997; Ray *et al.*, 2004; Zhang *et al. *2004). Herein, we report the structure of the title complex.

The title compound contains one Cu^II^ cation, one suc ligands, one bpm ligands, one coordinated water and two lattice water molecules, as illustrated in Fig. 1. The Cu^II^ atom has a slightly distored square-pyramidal geometry (Table 1), in which the Cu^II^atom is coordinated by two N atoms of bpm ligand, two O atoms from carboxyl groups of succinate anions and one O atom from coordinated water molecule. Each unit is connected by O—H···O hydrogen bonds between carboxyl groups and coordinated water molecules (Table 2) into one-dimensional chain along *c*-axis. The lattice water molecule acts as both hydrogen-bond donor and acceptor. Just through hydrogen bonds (O—H···O) involving lattice water molecules, those one-dimensional chains are further connected to generate a three-dimensional supramolecular framework.

### Experimental

A mixture of CuCl_2_.2H_2_O (0.017 g, 0.1 mmol), bpm (0.015 g, 0.1 mmol), sodium succinate (0.0139 g, 0.1 mmol) and distilled water (10 ml) was sealed in a 25 ml Teflon-lined stainless autoclave. The pH value of the mixture was adjusted to 6 by an aqueous solution of NaOH (0.1 mol/*L*), and then heated at 393 K for 3 days; blue crystals were obtained on cooling to room temperature at 5 K/h.

### Refinement

Water H atoms were located in a difference Fourier map and refined with distance restraints O—H = 0.85 (2) Å, *U*_iso_(H) = 1.5U_eq_(O). Other H atoms were placed in calculated positions and treated using a riding-model approximation with C—H = 0.93 Å and U_iso_(H) = 1.2U_eq_(C).

### Crystal data

**Table:**

[Cu(C_4_H_4_O_4_)(C_8_H_6_N_4_)(H_2_O)]·2H_2_O	*F*(000) = 804
*M**_r_* = 391.83	*D*_x_ = 1.691 Mg m^−^^3^
Monoclinic, *P*2_1_/*c*	Mo *K*α radiation, λ = 0.71073 Å
Hall symbol: -P 2ybc	Cell parameters from 3225 reflections
*a* = 10.6905 (8) Å	θ = 1.9–25.1°
*b* = 18.9321 (14) Å	µ = 1.46 mm^−^^1^
*c* = 7.6105 (6) Å	*T* = 293 K
β = 92.229 (1)°	Prism, blue
*V* = 1539.2 (2) Å^3^	0.30 × 0.20 × 0.09 mm
*Z* = 4	

### Data collection

**Table:**

Bruker SMART CCD diffractometer	2735 independent reflections
Radiation source: fine-focus sealed tube	2085 reflections with *I* > 2σ(*I*)
graphite	*R*_int_ = 0.036
φ and ω scans	θ_max_ = 25.1°, θ_min_ = 1.9°
Absorption correction: multi-scan (*SADABS*; Sheldrick, 1996)	*h* = −12→12
*T*_min_ = 0.700, *T*_max_ = 0.877	*k* = −22→22
7725 measured reflections	*l* = −9→4

### Refinement

**Table:**

Refinement on *F*^2^	Primary atom site location: structure-invariant direct methods
Least-squares matrix: full	Secondary atom site location: difference Fourier map
*R*[*F*^2^ > 2σ(*F*^2^)] = 0.043	Hydrogen site location: inferred from neighbouring sites
*wR*(*F*^2^) = 0.123	H atoms treated by a mixture of independent and constrained refinement
*S* = 1.06	*w* = 1/[σ^2^(*F*_o_^2^) + (0.0588*P*)^2^ + 1.4777*P*] where *P* = (*F*_o_^2^ + 2*F*_c_^2^)/3
2735 reflections	(Δ/σ)_max_ = 0.001
235 parameters	Δρ_max_ = 0.46 e Å^−^^3^
10 restraints	Δρ_min_ = −0.65 e Å^−^^3^

### Special details

**Table:**

Geometry. All e.s.d.'s (except the e.s.d. in the dihedral angle between two l.s. planes) are estimated using the full covariance matrix. The cell e.s.d.'s are taken into account individually in the estimation of e.s.d.'s in distances, angles and torsion angles; correlations between e.s.d.'s in cell parameters are only used when they are defined by crystal symmetry. An approximate (isotropic) treatment of cell e.s.d.'s is used for estimating e.s.d.'s involving l.s. planes.
Refinement. Refinement of *F*^2^ against ALL reflections. The weighted *R*-factor *wR* and goodness of fit *S* are based on *F*^2^, conventional *R*-factors *R* are based on *F*, with *F* set to zero for negative *F*^2^. The threshold expression of *F*^2^ > σ(*F*^2^) is used only for calculating *R*-factors(gt) *etc*. and is not relevant to the choice of reflections for refinement. *R*-factors based on *F*^2^ are statistically about twice as large as those based on *F*, and *R*- factors based on ALL data will be even larger.

### Fractional atomic coordinates and isotropic or equivalent isotropic displacement parameters (Å^2^)

**Table:**

	*x*	*y*	*z*	*U*_iso_*/*U*_eq_	
Cu1	0.83883 (5)	0.98092 (2)	0.42287 (7)	0.0376 (2)	
O1	0.7210 (3)	0.94912 (16)	0.1622 (4)	0.0477 (8)	
H1A	0.673 (4)	0.9136 (17)	0.160 (6)	0.072*	
H1B	0.774 (4)	0.945 (2)	0.082 (5)	0.072*	
O1W	0.6018 (4)	0.82533 (18)	0.2132 (6)	0.0724 (11)	
H1WA	0.5230 (12)	0.819 (3)	0.212 (9)	0.109*	
H1WB	0.637 (4)	0.790 (2)	0.170 (9)	0.109*	
O2	0.7834 (3)	1.07626 (14)	0.4543 (4)	0.0459 (7)	
O2W	0.6560 (4)	0.3145 (2)	0.2485 (6)	0.0810 (12)	
H2WB	0.674 (7)	0.2726 (15)	0.280 (8)	0.121*	
H2WA	0.676 (7)	0.321 (3)	0.143 (4)	0.121*	
O3	0.7344 (3)	1.18623 (16)	0.3913 (5)	0.0606 (9)	
O4	1.1035 (3)	1.06421 (15)	0.1176 (4)	0.0490 (8)	
O5	0.9903 (3)	1.00736 (14)	0.3069 (4)	0.0395 (7)	
N1	0.8883 (3)	0.87826 (17)	0.4388 (4)	0.0362 (8)	
N2	0.7034 (3)	0.94476 (18)	0.5757 (4)	0.0382 (8)	
N3	0.8133 (3)	0.76731 (17)	0.5315 (5)	0.0468 (9)	
N4	0.6295 (3)	0.8392 (2)	0.7042 (5)	0.0494 (10)	
C1	0.9872 (4)	0.8470 (2)	0.3680 (6)	0.0435 (10)	
H1C	1.0466	0.8745	0.3135	0.052*	
C2	1.0017 (4)	0.7748 (2)	0.3750 (6)	0.0499 (11)	
H2	1.0700	0.7525	0.3272	0.060*	
C3	0.9102 (4)	0.7374 (2)	0.4561 (6)	0.0491 (11)	
H3	0.9164	0.6884	0.4584	0.059*	
C4	0.8069 (4)	0.8370 (2)	0.5194 (5)	0.0368 (9)	
C5	0.7056 (4)	0.8751 (2)	0.6045 (5)	0.0376 (9)	
C6	0.5436 (4)	0.8772 (3)	0.7835 (7)	0.0573 (13)	
H6	0.4886	0.8539	0.8557	0.069*	
C7	0.5319 (4)	0.9492 (3)	0.7641 (6)	0.0546 (12)	
H7	0.4704	0.9746	0.8201	0.066*	
C8	0.6163 (4)	0.9822 (2)	0.6570 (6)	0.0459 (11)	
H8	0.6124	1.0309	0.6414	0.055*	
C9	0.7701 (4)	1.1270 (2)	0.3460 (6)	0.0413 (10)	
C10	0.7927 (4)	1.1167 (2)	0.1584 (6)	0.0442 (11)	
H10A	0.7179	1.0939	0.1097	0.053*	
H10B	0.7932	1.1638	0.1082	0.053*	
C11	0.8904 (4)	1.0823 (2)	0.0863 (6)	0.0409 (10)	
H11A	0.9243	1.1155	0.0036	0.049*	
H11B	0.8523	1.0448	0.0156	0.049*	
C12	1.0022 (4)	1.0491 (2)	0.1772 (5)	0.0370 (9)	

### Atomic displacement parameters (Å^2^)

**Table:**

	*U*^11^	*U*^22^	*U*^33^	*U*^12^	*U*^13^	*U*^23^
Cu1	0.0438 (3)	0.0255 (3)	0.0444 (3)	0.0014 (2)	0.0122 (2)	0.0028 (2)
O1	0.0475 (18)	0.0452 (17)	0.0511 (19)	−0.0081 (14)	0.0088 (15)	−0.0032 (15)
O1W	0.071 (2)	0.0425 (19)	0.104 (3)	−0.0055 (18)	0.012 (2)	−0.008 (2)
O2	0.0617 (19)	0.0287 (14)	0.0479 (18)	0.0054 (14)	0.0112 (15)	0.0025 (13)
O2W	0.100 (3)	0.063 (2)	0.081 (3)	0.028 (2)	0.022 (3)	0.024 (2)
O3	0.083 (2)	0.0362 (17)	0.063 (2)	0.0214 (17)	0.0051 (19)	−0.0003 (16)
O4	0.0475 (18)	0.0434 (17)	0.057 (2)	−0.0044 (14)	0.0187 (16)	0.0048 (15)
O5	0.0417 (16)	0.0332 (14)	0.0439 (17)	−0.0001 (12)	0.0055 (13)	0.0063 (13)
N1	0.0393 (18)	0.0300 (17)	0.0395 (19)	0.0004 (15)	0.0026 (16)	−0.0003 (15)
N2	0.0403 (18)	0.0358 (18)	0.039 (2)	0.0019 (15)	0.0066 (16)	0.0009 (15)
N3	0.055 (2)	0.0281 (18)	0.057 (2)	−0.0029 (16)	0.001 (2)	0.0058 (17)
N4	0.042 (2)	0.049 (2)	0.058 (2)	−0.0074 (17)	0.0105 (19)	0.0130 (19)
C1	0.046 (2)	0.041 (2)	0.044 (2)	−0.001 (2)	0.008 (2)	−0.002 (2)
C2	0.052 (3)	0.041 (2)	0.057 (3)	0.010 (2)	0.005 (2)	−0.003 (2)
C3	0.062 (3)	0.030 (2)	0.056 (3)	0.006 (2)	0.003 (2)	−0.001 (2)
C4	0.038 (2)	0.033 (2)	0.039 (2)	−0.0051 (17)	0.0001 (19)	0.0064 (18)
C5	0.038 (2)	0.037 (2)	0.038 (2)	−0.0028 (18)	−0.0011 (19)	0.0060 (18)
C6	0.045 (3)	0.069 (3)	0.059 (3)	−0.003 (2)	0.011 (2)	0.014 (3)
C7	0.045 (3)	0.068 (3)	0.051 (3)	0.005 (2)	0.012 (2)	0.002 (3)
C8	0.050 (3)	0.042 (2)	0.046 (3)	0.010 (2)	0.005 (2)	0.002 (2)
C9	0.038 (2)	0.032 (2)	0.054 (3)	0.0007 (18)	0.006 (2)	0.001 (2)
C10	0.044 (2)	0.033 (2)	0.056 (3)	−0.0061 (19)	0.001 (2)	0.010 (2)
C11	0.050 (2)	0.031 (2)	0.042 (2)	−0.0139 (19)	0.003 (2)	0.0073 (19)
C12	0.048 (2)	0.0251 (19)	0.039 (2)	−0.0006 (18)	0.008 (2)	−0.0044 (18)

### Geometric parameters (Å, °)

**Table:**

Cu1—O1	2.386 (3)	N4—C5	1.323 (5)
Cu1—O2	1.918 (3)	N4—C6	1.330 (6)
Cu1—O5	1.940 (3)	C1—C2	1.376 (6)
Cu1—N1	2.017 (3)	C1—H1C	0.9300
Cu1—N2	2.012 (3)	C2—C3	1.374 (6)
O1—H1A	0.85 (4)	C2—H2	0.9300
O1—H1B	0.85 (4)	C3—H3	0.9300
O1W—H1WA	0.85 (4)	C4—C5	1.472 (6)
O1W—H1WB	0.84 (4)	C6—C7	1.377 (7)
O2—C9	1.270 (5)	C6—H6	0.9300
O2W—H2WB	0.85 (4)	C7—C8	1.388 (6)
O2W—H2WA	0.85 (4)	C7—H7	0.9300
O3—C9	1.239 (5)	C8—H8	0.9300
O4—C12	1.224 (5)	C9—C10	1.469 (6)
O5—C12	1.275 (5)	C10—C11	1.365 (5)
N1—C4	1.336 (5)	C10—H10A	0.9700
N1—C1	1.343 (5)	C10—H10B	0.9700
N2—C5	1.336 (5)	C11—C12	1.495 (6)
N2—C8	1.341 (5)	C11—H11A	0.9700
N3—C4	1.324 (5)	C11—H11B	0.9700
N3—C3	1.330 (5)		
			
O2—Cu1—O5	94.68 (12)	N3—C4—N1	125.6 (4)
O2—Cu1—N2	90.87 (13)	N3—C4—C5	119.7 (3)
O5—Cu1—N2	169.41 (13)	N1—C4—C5	114.7 (3)
O2—Cu1—N1	168.87 (13)	N4—C5—N2	126.5 (4)
O5—Cu1—N1	93.13 (13)	N4—C5—C4	118.6 (4)
N2—Cu1—N1	80.22 (13)	N2—C5—C4	114.9 (3)
O2—Cu1—O1	100.69 (12)	N4—C6—C7	123.2 (4)
O5—Cu1—O1	96.32 (12)	N4—C6—H6	118.4
N2—Cu1—O1	91.50 (12)	C7—C6—H6	118.4
N1—Cu1—O1	86.30 (12)	C6—C7—C8	116.8 (4)
Cu1—O1—H1A	121 (4)	C6—C7—H7	121.6
Cu1—O1—H1B	106 (3)	C8—C7—H7	121.6
H1A—O1—H1B	109 (4)	N2—C8—C7	120.8 (4)
H1WA—O1W—H1WB	110 (5)	N2—C8—H8	119.6
C9—O2—Cu1	131.1 (3)	C7—C8—H8	119.6
H2WB—O2W—H2WA	110 (6)	O3—C9—O2	122.1 (4)
C12—O5—Cu1	128.5 (3)	O3—C9—C10	117.0 (4)
C4—N1—C1	117.6 (3)	O2—C9—C10	120.9 (4)
C4—N1—Cu1	114.7 (3)	C11—C10—C9	127.7 (4)
C1—N1—Cu1	127.5 (3)	C11—C10—H10A	105.4
C5—N2—C8	117.0 (4)	C9—C10—H10A	105.4
C5—N2—Cu1	115.0 (3)	C11—C10—H10B	105.4
C8—N2—Cu1	128.0 (3)	C9—C10—H10B	105.4
C4—N3—C3	115.7 (4)	H10A—C10—H10B	106.0
C5—N4—C6	115.7 (4)	C10—C11—C12	128.7 (4)
N1—C1—C2	120.7 (4)	C10—C11—H11A	105.1
N1—C1—H1C	119.6	C12—C11—H11A	105.1
C2—C1—H1C	119.6	C10—C11—H11B	105.1
C3—C2—C1	116.7 (4)	C12—C11—H11B	105.1
C3—C2—H2	121.7	H11A—C11—H11B	105.9
C1—C2—H2	121.7	O4—C12—O5	123.2 (4)
N3—C3—C2	123.6 (4)	O4—C12—C11	115.7 (4)
N3—C3—H3	118.2	O5—C12—C11	121.1 (4)
C2—C3—H3	118.2		
			
O5—Cu1—O2—C9	51.3 (4)	C3—N3—C4—C5	−177.7 (4)
N2—Cu1—O2—C9	−137.8 (4)	C1—N1—C4—N3	−2.0 (6)
N1—Cu1—O2—C9	−174.3 (6)	Cu1—N1—C4—N3	173.5 (4)
O1—Cu1—O2—C9	−46.1 (4)	C1—N1—C4—C5	176.0 (4)
O2—Cu1—O5—C12	−50.4 (3)	Cu1—N1—C4—C5	−8.5 (4)
N2—Cu1—O5—C12	−171.8 (6)	C6—N4—C5—N2	−0.5 (7)
N1—Cu1—O5—C12	137.5 (3)	C6—N4—C5—C4	177.1 (4)
O1—Cu1—O5—C12	50.9 (3)	C8—N2—C5—N4	0.7 (6)
O2—Cu1—N1—C4	43.9 (8)	Cu1—N2—C5—N4	177.8 (3)
O5—Cu1—N1—C4	178.4 (3)	C8—N2—C5—C4	−177.1 (4)
N2—Cu1—N1—C4	6.7 (3)	Cu1—N2—C5—C4	0.0 (5)
O1—Cu1—N1—C4	−85.5 (3)	N3—C4—C5—N4	5.8 (6)
O2—Cu1—N1—C1	−141.1 (6)	N1—C4—C5—N4	−172.3 (4)
O5—Cu1—N1—C1	−6.6 (4)	N3—C4—C5—N2	−176.3 (4)
N2—Cu1—N1—C1	−178.3 (4)	N1—C4—C5—N2	5.6 (5)
O1—Cu1—N1—C1	89.5 (4)	C5—N4—C6—C7	0.5 (7)
O2—Cu1—N2—C5	−176.8 (3)	N4—C6—C7—C8	−0.6 (8)
O5—Cu1—N2—C5	−55.1 (8)	C5—N2—C8—C7	−0.8 (6)
N1—Cu1—N2—C5	−3.5 (3)	Cu1—N2—C8—C7	−177.4 (3)
O1—Cu1—N2—C5	82.5 (3)	C6—C7—C8—N2	0.8 (7)
O2—Cu1—N2—C8	0.0 (4)	Cu1—O2—C9—O3	−178.8 (3)
O5—Cu1—N2—C8	121.6 (7)	Cu1—O2—C9—C10	3.2 (6)
N1—Cu1—N2—C8	173.2 (4)	O3—C9—C10—C11	137.1 (5)
O1—Cu1—N2—C8	−100.8 (4)	O2—C9—C10—C11	−44.9 (7)
C4—N1—C1—C2	1.6 (6)	C9—C10—C11—C12	−3.2 (7)
Cu1—N1—C1—C2	−173.3 (3)	Cu1—O5—C12—O4	177.3 (3)
N1—C1—C2—C3	0.5 (7)	Cu1—O5—C12—C11	−3.5 (5)
C4—N3—C3—C2	2.2 (7)	C10—C11—C12—O4	−131.7 (4)
C1—C2—C3—N3	−2.5 (7)	C10—C11—C12—O5	49.0 (6)
C3—N3—C4—N1	0.2 (6)		

### Hydrogen-bond geometry (Å, °)

**Table:**

*D*—H···*A*	*D*—H	H···*A*	*D*···*A*	*D*—H···*A*
O1—H1A···O1W	0.85 (4)	1.89 (4)	2.703 (5)	162 (4)
O1—H1B···O4^i^	0.85 (4)	2.05 (4)	2.903 (4)	178 (4)
O1W—H1WA···O2W^ii^	0.85 (4)	1.95 (2)	2.790 (6)	169 (5)
O1W—H1WB···N3^iii^	0.84 (4)	2.45 (5)	3.216 (5)	152 (4)
O1W—H1WB···N4^iii^	0.84 (4)	2.46 (4)	3.130 (5)	137 (5)
O2W—H2WA···O3^iii^	0.85 (4)	2.04 (4)	2.876 (6)	167 (5)
O2W—H2WB···O3^iv^	0.85 (4)	1.94 (4)	2.777 (5)	168 (4)

Symmetry codes: (i) −*x*+2, −*y*+2, −*z*; (ii) −*x*+1, *y*+1/2, −*z*+1/2; (iii) *x*, −*y*+3/2, *z*−1/2; (iv) *x*, *y*−1, *z*.
